# Starting Them Young: Introducing High School Students to Community Gerontology

**DOI:** 10.1093/geroni/igaa057.026

**Published:** 2020-12-16

**Authors:** Marjorie Getz, Sherri Morris

**Affiliations:** 1 Methodist College, Peoria, Illinois, United States; 2 Bradley University, Peoria, Illinois, United States

## Abstract

The Clinical Research Experience Internship Program (CREST) provides participants with foundations in scientific research appropriate for high school students interested in clinical careers in health-related disciplines (for example, nursing). The overall program goal is to provide research and career experiences to individuals from disadvantaged backgrounds, racial/ethnic minorities, and others who are underrepresented in these fields. The focus of this presentation is that part of the CREST program that has been ongoing since 2013. One program mentor has provided an internship experience to 22 high school students training in and work experience with community-based programs designed to improve health for older adults (identified as ‘community gerontology’). This poster presentation describes some of these experiences (e.g., preparation of caregiver support materials, preparation of nutrition based materials for congregate meal sites for older adults, coaches’ training and program implementation of several evidence-based community programs). Students have worked with older adults in senior housing facilities, supported housing complexes for veterans, and congregate meal sites for older adults. Because of the program experience, the CREST program helps dispel common stereotypes about older adults and encourages students exploring possible clinical career options to consider focusing on older adults as client populations. Program components are described which can allow conference participants to decide on the applicability of this type of programming for their own communities. Qualitative data are presented that provide insights into these experiences as these influence choice of college major and projected career paths and attitudes about working in community-based healthcare with older people.

